# Ascorbic Acid and the Premature Infant

**DOI:** 10.3390/nu14112189

**Published:** 2022-05-24

**Authors:** Nayef Chahin, Miheret S. Yitayew, Alicia Richards, Brielle Forsthoffer, Jie Xu, Karen D. Hendricks-Muñoz

**Affiliations:** 1 Division of Neonatal Medicine, Department of Pediatrics, Children’s Hospital of Richmond at VCU and School of Medicine, Virginia Commonwealth University, Richmond, VA 23298-02761, USA; miheret.yitayew@vcuhealth.org (M.S.Y.); jie.xu@vcuhealth.org (J.X.); karen.hendricks-munoz@vcuhealth.org (K.D.H.-M.); 2 Department of Biostatistics, Virginia Commonwealth University, Richmond, VA 23298-02761, USA; richardsar@vcu.edu (A.R.); forsthofferba@mymail.vcu.edu (B.F.)

**Keywords:** vitamin C, ascorbic acid, prematurity, premature nutrition

## Abstract

Little information exists about the plasma target nutritional needs of the >15 million premature infants <37 weeks gestation. Investigating ascorbic acid’s (AscA) role in infant health, our study details the relationship of infant characteristics and maternal health on infant plasma AscA level (pAscA) during postnatal development. Furthermore, we determined pAscA influence during the first week of life (EpAscA) with later infant morbidities. We hypothesize that pAscA is influenced by gestational organ immaturity, as well as maternal factors, with EpAscA associated with greater morbidity risk. We conducted a prospective longitudinal observational study of pAscA, demographics and hospital course detailed in infants ≤34 weeks. Sixty-three subjects were included, with >200 urine and plasma data points analyzed. Maternal smoking, exposure to magnesium sulfate (MgSO4) and advancing gestational and postnatal age were associated with lower pAscA. Non-white infants and those ≤30 weeks that developed bronchopulmonary dysplasia or retinopathy of prematurity had lower pAscA. Prenatal smoking, MgSO4, birth gestational age and race negatively influence pAscA. These results show prenatal and postnatal developmental factors influencing initial pAscA and metabolism, potentially setting the stage for organ health and risk for disease. Assessment of dietary targets may need adjustment in this population.

## 1. Introduction

Ascorbic acid (AscA), commonly known as vitamin C, is a water-soluble vitamin, antioxidant and immune-response enhancer essential for human survival, commonly associated with the development of scurvy and its complications if AscA-deficient [1,2,3,4,5,6]. Due to their inability to produce AscA, humans entirely rely on external sources, mainly diet, to maintain adequate plasma levels and prevent a state of deficiency [7,8,9]. This becomes of utmost importance to neonates, who completely rely on the active transfer of AscA in utero and postnatally through breastmilk amongst other sources [10,11,12,13,14].

Initial observational studies assessing plasma AscA (pAscA) in neonates have reported higher cord-blood levels of pAscA measured in healthy-term newborns at birth compared to their mothers, followed by a significant drop in plasma concentrations over the first 24 h of life [15,16,17], suggesting an active transport system within the placenta to preferentially increase circulating AscA in the developing infant that is postnatally dissociated. Likewise, higher cord-blood levels in premature infants compared to those at term have been identified (146 ± 93 μM/L vs. 102 ± 27 μM/L, respectively; *p* = 0.03), with a rapid decrease seen over a few days in preterm infants, which suggests a continued need for AscA supplementation in preterm infants [18,19,20]. These studies found the need for further understanding of supplementation requirements, particularly for preterm infants with increasing survival at the lowest gestational age of viability. Few reports have investigated the influence of maternal health, gestational age, body mass or risk of morbidity development based on pAscA concentrations during the postnatal maturation of premature infants. Silvers et al. through a prospective longitudinal observational study reported significantly higher concentrations of pAscA within two hours of life in premature infants who died vs. those who survived. An increased risk of bronchopulmonary dysplasia (BPD) was also seen with higher pAscA levels measured on day 2 of life [20]. On the contrary, lower pAscA levels measured at 10 days of life (Mosion, RM) and at 28 days of life (Sluis et al.) were seen in premature infants who developed BPD [21,22]. Subsequently, a randomized control trial comparing the combined administration of parenteral and enteral low-dose AscA (10 mg/kg/day) vs. high dose (30–40 mg/kg/day) vs. placebo to premature infants reported no significant difference in clinical outcome amongst the treatment groups; although no levels were identified, these data suggested improved respiratory outcome in those who received high-dose AscA [23]. Similarly, studies have demonstrated a relationship between maternal smoking and breastmilk AscA levels as well as maternal smoking and offspring pAscA levels in relation to newborn pulmonary function test results, with higher pAscA levels found in breastmilk and offspring when mothers were supplemented with AscA [24,25].

Current recommendations suggest that premature infants receive higher doses of AscA (25–31 mg/kg/day) to approximate concentrations seen in the third trimester in utero, with pAscA levels of <15 mg/dL associated with deficiency as per adult and term infant data [14,26,27]. This creates a challenge, as most initially receive AscA parenterally or enterally through multivitamin supplements that can increase other nutrients unintentionally. There are limited data available to guide optimal plasma concentrations postnatally by gestational age or during sickness. Additionally, the diagnosis of scurvy is rare in children and may be uniquely challenged in extremely preterm infants, yet it is this population that is at a high risk for inflammatory processes and/or mortality [28].

In this study, we aim to detail the relationship of gestational age (GA), birthweight, race, maternal health and evolving preterm hepatic and renal maturation on infant pAscA levels during infant development in the neonatal intensive care unit (NICU). Furthermore, we detail pAscA levels on influencing later preterm-infant common morbidities (bronchopulmonary dysplasia (BPD), infection, necrotizing enterocolitis (NEC), retinopathy of prematurity (ROP) and intraventricular hemorrhage (IVH)) in infants ≤34 weeks of gestation, where we hypothesize that lower-than-expected pAscA levels are associated with greater morbidity risk.

## 2. Materials and Methods

### 2.1. Study Design and Participants

This prospective longitudinal observational study was performed at the Children’s Hospital of Richmond at Virginia Commonwealth University (VCU) from July 2015–Jan 2019. Approval for the study was obtained by the Institutional Review Board at VCU, and parental written consent was obtained during prenatal counseling for prematurity or within 24 h of birth. Inborn preterm infants (gestational age ≤ 34 weeks) admitted to the neonatal intensive care unit (NICU) were eligible for study inclusion. Outborn preterm infants, infants >34 weeks of gestation, infants with severe congenital anomalies and infants who had no written consent were excluded. All subjects enrolled took part until the day of discharge, death or parental request to be withdrawn from the study. No subjects were withdrawn from the study.

### 2.2. Clinical Data and Outcomes

Clinical data were collected using our electronic medical records and included the following: subject demographic characteristics (gestational age (GA), birthweight (BW), small for gestational age (SGA) (<10th percentile) status, gender, race, postmenstrual age (PMA), parenteral (TPN) and enteral nutrition provided (breastmilk and/or pasteurized donor human milk (PDHM) vs. formula), and maternal characteristics such as age, health condition, medications taken, smoking status and use of prenatal vitamins. BPD, or a composite of BPD and death as a secondary outcome, was documented and defined as per the National Institute of Health/National Institute of Child Health and Human Development: need of oxygen for at least 28 days, with severity determined at 36 weeks (PMA) or at discharge, whichever came first, as the following: mild if breathing room air; moderate if requiring <30% fraction of inspired oxygen; severe if requiring ≥30% fraction of inspired oxygen or need for positive pressure support [29,30,31,32]. Other outcomes collected included sepsis defined as a proven positive blood culture; necrotizing enterocolitis (NEC) stage 2 or greater as per modified Bell’s criteria; intraventricular hemorrhage (IVH) diagnosed using transfontanelle ultrasonography obtained on the 7th day of life as per unit practice or earlier if clinically indicated and classified based on Papile’s grading system [33]; and retinopathy of prematurity (ROP) staging based on the International Classification of Retinopathy of Prematurity (ICROP) [34].

### 2.3. Plasma Ascorbic-Acid Collection and Processing

Cord blood was collected when possible postdelivery of placenta and first-day samples were obtained, with first assessments performed at time of admission to the NICU if no cord blood was obtained. The rest of the blood samples were obtained weekly thereafter during routine monitoring of the nutritional status of the preterm infant and during assessments conducted for acute illness. No blood samples were obtained out of the period and circumstances previously specified. Blood was collected in EDTA-treated tubes, shielded from light, dated, timed and transported from the NICU to the laboratory on ice and then centrifuged at 2000× *g* for 10 min at 4 °C. The plasma was carefully separated from the cells and deproteinized as follows: 100 µL of plasma was treated with 200 µL of cold 20% trichloroacetic acid (TCA) to deproteinize, and 200 µL of cold 0.2% dithiothreitol (DTT) to prevent AscA oxidation. The mixture was kept on ice, vortexed intermittently for 2 min and centrifuged at 10,000× *g*, 4 °C, for 10 min. The supernatant fraction was transferred to a fresh tube and stored at −80 °C for batch analysis using a fluorescence end-point assay. The AscA assay was conducted in duplicate in a black flat-bottom 96-well plate with date and time of sample processing recorded. The assay reaction consisted of 50 μL of either standard AscA or sample and 200 μL of assay buffer containing 1 M sodium acetate, pH 5.5, 1 mM Tempol and 1 mM o-phenylenediamine. After 30 min incubation at room temperature in the dark, fluorescence intensity was measured at 345 nm excitation and 425 nm emission with Tecan Safire2, multifunction monochromator plate reader. The concentration of AscA in the sample was estimated using linear regression of the known standard concentration [35].

### 2.4. Urine Ascorbic-Acid Collection and Analysis

Infant’s urine was captured with cotton balls placed in the diaper during diaper change, and urine specimen was recovered by squeezing soaked cotton balls with a 10 mL syringe into a collection tube. Samples were transported from the clinic to laboratory on ice. Urine samples were centrifuged at 10,000× *g* for 10 min at 4 °C to clarify and remove any insoluble material and put into aliquots and stored at −80 °C for batch analysis using a fluorescence end-point assay. The ascorbic-acid assay was conducted in duplicate in a black flat-bottom 96-well plate. Urine samples were diluted 26-fold with DI water prior to analysis due to its higher ascorbic-acid concentration in general. The assay reaction mixture was consisted of 50 μL of either standard ascorbic acid or sample and 200 μL of assay buffer containing 1 M sodium acetate, pH 5.5, 1 mM Tempol and 1 mM o-phenylenediamine. After 30 min incubation at room temperature in the dark, fluorescence intensity was measured at 345 nm excitation and 425 nm emission with Tecan Safire2, multifunction monochromator plate reader. The concentration of the ascorbic acid in the sample was estimated using linear regression of the known standard concentration. For samples that yielded higher or lower fluorescent readings beyond standard curve range, analyses were repeated with dilutions accordingly [35].

### 2.5. Statistical Analysis

Subject characteristics were summarized overall as frequencies and percentages or means and standard deviations. Various linear mixed models—unadjusted and adjusted—for the subject predictions were used to model AscA levels, with a random effect for subject. Models using both the plasma and cord-blood samples and using only the plasma samples were conducted. Some models only analyzed data from the first week of observation for each patient while others looked at all the data for each patient. For each model, the difference in the AscA levels (plasma and urine), standard error of the difference, 95% confidence interval and *p*-value were reported. A sub analysis of those ≤30 weeks of gestation was performed due to their inherent higher risk for inflammatory processes [36]. All analyses were performed at a statistical significance level of 0.05 using SAS Version 9.4 Statistical Software (SAS Institute, Cary, NC, USA).

## 3. Results

### 3.1. Infant Demographics

Sixty-three premature infants were included in the study with a mean GA of 28.4 ± 3 weeks, of which 20.6% were small for gestational age <10 percentile. Racial ethnic composition included 56% black, 31% white and 11.1% Hispanic. Gender included 51% male and 49% female (Table 1). The mean length of stay was 85.2 days SD (50.4), 19% developed moderate-to-severe BPD, 29% had at least one blood-culture-proven infectious episode and 6 infants (9%) died during the study period (Table 2).

A total of 325 plasma samples (inclusive of cord blood) and 201 urine samples were collected and analyzed throughout the study period, with a median of 5 (1–17) plasma and 3 (1–11) urine samples collected per patient.

### 3.2. Plasma and Urine Ascorbic Acid Levels

The initial mean AscA plasma level (pAscA) at time of enrollment was 1.5 mg/dL, SD (0.7), with a statistically significant decrease in the mean pAscA level noted with advancing postanal age in days regardless of corrected gestational age (1.2 mg/dL, SD (0.8), *p* = 0.002). Of the 63 premature infants, 53 (84%) had a first-week-of-life sample, with a mean GA of 28.0 weeks, SD (2.9) and mean birthweight (BW) of 1139.8 g, SD (407.8). The mean AscA plasma level (pAscA) was 1.45 mg/dL, SD (0.72) and mean AscA urine level (uAscA) was 17.8 mg/dL, SD (22) (Table 3). Urinary AscA levels increased with increasing postnatal age in weeks as pAscA levels declined.

Overall patient characteristics, morbidities and mean plasma and urine AscA levels are described in Table 1, Table 2 and Table 3.

### 3.3. Plasma Ascorbic-Acid Level in Relation to Race, Gestational Age and Birthweight

There were associations between early pAscA levels (≤7 days of life) in relation to infant/maternal race and infant GA. White infants presented overall higher pAscA levels compared to black and Hispanic infants. Furthermore, when adjusting for race, SGA status, and birthweight, as GA increased by 1-week AscA levels decreased by 0.16 mg/dL (*p*-value = 0.0246, 95% CI: −0.30, −0.03) (Table 4). No associations were found between pAscA levels and birthweight, changing infant weight over the study period or SGA status (not shown).

### 3.4. Plasma Ascorbic-Acid Levels in Relation to Type of Nutrition Provided

When we adjusted for type of nutrition provided (enteral vs. parental, breastmilk vs. formula), birthweight, and SGA status, only gestational age was associated with pAscA levels (*p* = 0.0185, 95% CI: −0.35, −0.04) (Table 5).

### 3.5. Plasma Ascorbic-Acid Levels in Very Preterm Infants ≤30 Weeks

In a subanalysis of premature infants ≤30 weeks of gestation, 68% of infants were black and 55% male. For the total cohort, the mean pAscA level was 1.5 mg/dL SD (0.74), and the mean urine AscA level of 24 mg/dL SD (22). Mean plasma creatinine obtained for renal-function assessment at time of urine-sample collection was 0.56 ± 0.25 SD mg/dL and the mean aspartate aminotransferase, alanine aminotransferase and direct bilirubin obtained for liver-function assessment at time of plasma-sample collection was 59 mg/dL SD (51), 15 mg/dL SD (38) and 0.6 mg/dL SD (0.6), respectively (Table 6).

### 3.6. Plasma Ascorbic-Acid Level and Risk for Morbidity

**Figure 1:**
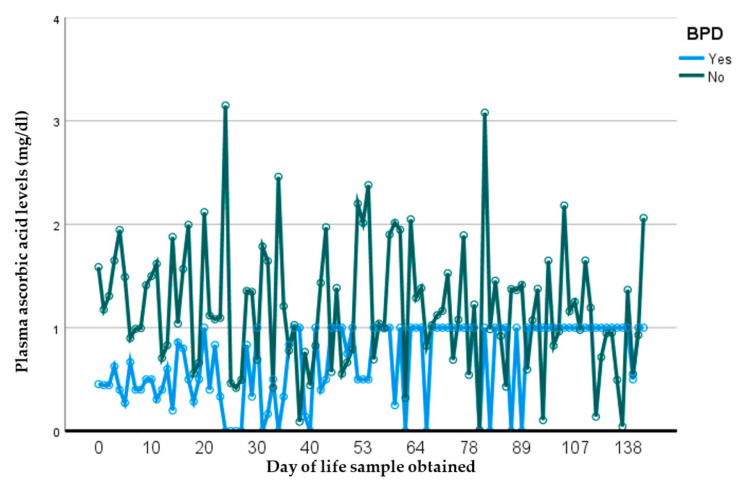
Measured plasma ascorbic-acid levels (mg/dL) per day of life in preterm infants ≤34 weeks with and without BPD. Infants who developed BPD had significantly lower pAscA levels at birth and these remained low throughout the study period compared to those who did not develop BPD (*p* = 0.0003, 95% CI = −0.36, 2.76). BPD = bronchopulmonary dysplasia, pAscA = plasma ascorbic acid.

**Figure 2:**
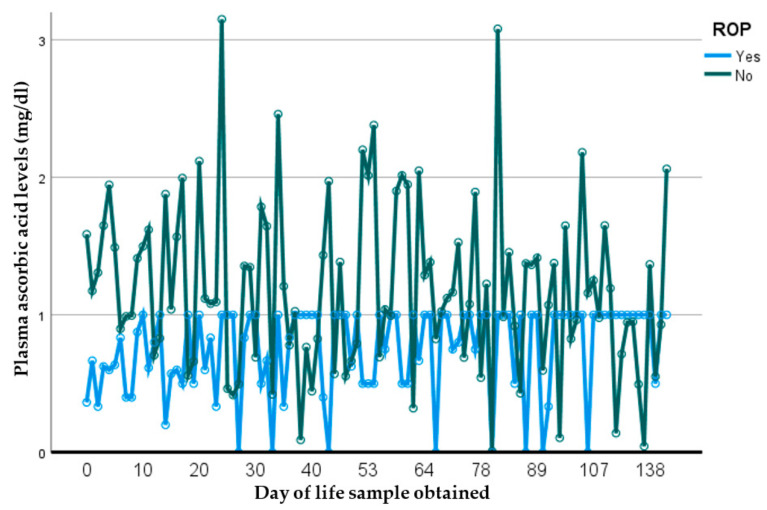
Measured plasma ascorbic-acid levels (mg/dL) per day of life in preterm infants ≤34 weeks with and without ROP. Infants who developed any ROP had significantly lower pAscA levels at birth and these remained low throughout the study period compared to those who did not develop ROP (*p* = 0.029, 95% CI: −0.43, 2.63). ROP = retinopathy of prematurity, pAscA= plasma ascorbic acid.

To assess the influence of early pAscA levels on later morbidity, a subset of 47 infants with early-life first-week samples were analyzed. Of these, 17 (37%) developed moderate-to-severe BPD, 18 (38%) had at least one infectious episode (Table 6) and 2 (4%) died (not shown). There was no difference in the mean pAscA level in those who died (1.49 mg/dL, SD (0.72) vs. 1.39 mg/dL, SD (0.7), *p* = 0.06). The first pAscA level at birth of those who developed BPD was significantly lower and remained low throughout the study period compared to those who did not develop BPD (1.3 mg/dL vs. 2.0 mg/dL, *p* = 0.0003, 95% CI = −0.36, 2.76) (Figure 1). Additionally, those who developed moderate-to-severe BPD showed even lower levels of pAscA (0.9 mg/dL compared to 2.0 mg/dL, *p*= 0.04, 95% CI −0.63, 2.42) (not shown). When exploring the risk of developing other comorbidities (Table 7), mean pAscA levels were lower in premature infants who developed any retinopathy of prematurity (ROP) compared to those who did not (1.1 mg/dL vs. 1.4, *p* = 0.029, 95% CI: −0.43, 2.63) (Figure 2). There was no association of pAscA with greater risk of any stage IVH, NEC or later nosocomial infection or sepsis in the cohort.

### 3.7. Plasma Ascorbic-Acid Levels in Relation to Maternal Illness

When exploring the association between maternal factors and infant mean pAscA levels, reported maternal smoking was associated with higher mean pAscA levels compared to those who did not report smoking (1.6 mg/dL vs. 1.18 mg/dL, *p* = 0.012, 95% CI 0, 3.2) (not shown). Similarly, maternal administration of magnesium sulfate was associated with infant increased pAscA (1.4 mg/dL vs. 1.03 mg/dL, *p* = 0.00019, 95% CI −0.2, 3) (not shown). Other maternal illnesses evaluated included chorioamnionitis, preeclampsia, hypertension and medication use without associated alterations in pAscA levels in the infants (not shown).

### 3.8. Plasma Ascorbic-Acid Levels in Relation to Infant Gestational Maturity

**Figure 3:**
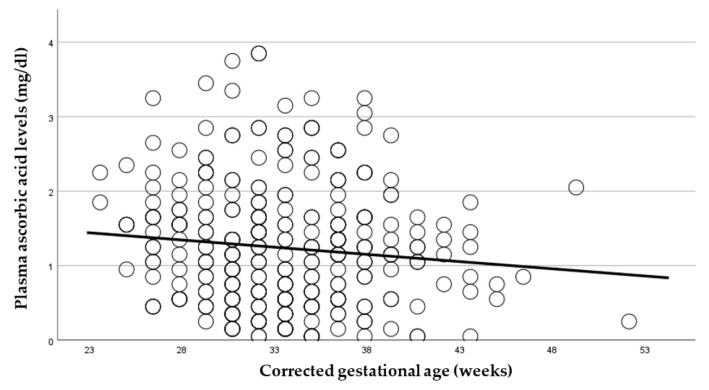
Measured plasma ascorbic-acid levels (mg/dL) in relation to corrected gestational age (weeks). Plasma ascorbic-acid levels were significantly lower with advancing preterm infant maturity outside the maternal womb (corrected gestational age) regardless of gestational age at birth (*p* < 0.027).

**Figure 4:**
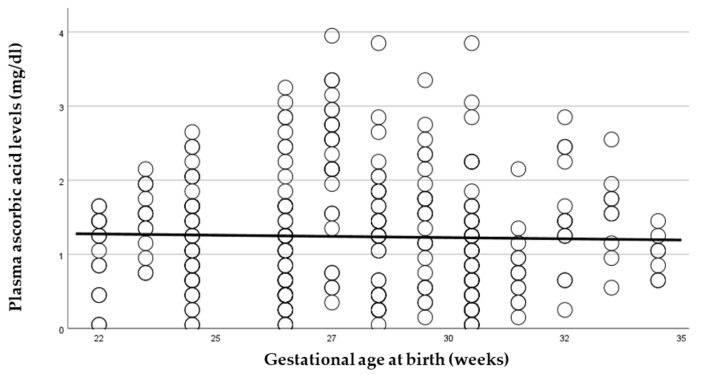
Measured plasma ascorbic-acid levels (mg/dL) in relation to gestational age at birth (weeks). With increased gestational age at birth, plasma ascorbic-acid levels were significantly lower (*p* < 0.001).

As pAscA metabolism of the infant is dependent on advancing renal function, we determined the influence of gestational-age maturity inside the womb compared to outside the womb on pAscA levels in this preterm population. Advancing gestational age (GA) outside the womb regardless of the GA at birth was associated with declining pAscA, *p* < 0.027 (Figure 3). Advancing GA by week at birth was also associated with lower pAscA, suggesting that the ex utero and in utero maturational processes governing the metabolism of ascorbic acid were similar and associated with gestational maturity of the infant (*p* < 0.001) (Figure 4).

## 4. Discussion

In this prospective observational longitudinal study, we address current gaps that exist to outline the important relationship between plasma ascorbic acid (pAscA) and premature infant health and disease. To our knowledge, there is limited information regarding pAscA levels in premature infants at birth and longitudinally, to address current normative levels for nutrient requirements in this at-risk population. Additionally, earlier information is not relevant to be inclusive of extremely preterm infants and micro preemies, with no studies showing the potential influence of sociodemographic racial determinants of maternal health on infant pAscA levels as well as risk for morbidity outcomes in premature infants. As seen with earlier studies, we found that as gestational age increases, pAscA levels decrease in the first week of life for preterm infants and continues to decrease with increasing gestational age. However, we saw that early and later AscA levels were associated with specific later severe chronic lung disease (BPD) and retinopathy of prematurity. We noted no associations of AscA levels in later infections, IVH or NEC. However, in those preterm infants who developed BPD, we noted that pAscA levels started at lower “normal” normative levels that remained lower throughout the study period compared to those who did not develop BPD. We believe this finding to be important, as it could suggest that an already immature anti-inflammatory system could be worsened by an ineffective antioxidant response, and that expected normative levels of ascorbic acid may be insufficient in infants at risk of BPD. This relationship was noted by Vyas et al., who assessed bronchoalveolar lavage fluid (BALF) ascorbate levels and risk for BPD in premature infants, reporting a delayed increase in BALF ascorbate levels to be associated with an increased risk for BPD, most likely due to a lack of antioxidant protection [37]. Furthermore, it could find that certain medications provided to infants with developing chronic lung disease may influence circulating AscA levels. Similarly, through its antioxidant effect, ascorbic acid could play a role in the prevention of ROP, with data being controversial and scarce [38]. To our knowledge, this is the first study to report ascorbic-acid levels in premature infants, specifically those ≤30 weeks and risk for ROP.

The observed higher levels of pAscA at birth and/or in the first day of life aligns with those observations made by Awoyelu et al. [15] and Silvers et al. [20]. Given the dependency of active transport of AscA through the placenta to maintain physiologic levels in the fetus, these results suggest that mothers in our studies consumed prenatal vitamins during the perinatal period and highlight the importance of maternal nutritional status during pregnancy as a significant influence on fetal AscA levels. This protective phenomenon persists in the postnatal life, where maternal health status and dietary intake of AscA influences its availability in breastmilk and is another avenue of communication to advance levels of AscA in infants of breastfeeding mothers. Although not reported in our study, breastmilk AscA levels measured in our lab were higher in colostrum, with a decrease in levels over the course of lactation as described by others [14,39]. Another major observation from our study focused on racial differences among black, white and Hispanic infants in both the first week of life and over the entire study period. The striking increase in plasma AscA levels in white infants at birth is likely due to maternal differences in prenatal vitamin intake related to social determinants of health, although genetic variant predispositions affecting the function of the sodium-dependent AscA transporters in the body and/or the antioxidant response in times of growth and disease cannot be excluded [40,41,42]. Additionally, our study uncovered that maternal exposure to perinatal magnesium and prenatal smoking impacted infant pAscA levels, as seen by McEvoy et al. [25], while the burden of other maternal diseases such as preeclampsia, hypertension, chorioamnionitis and maternal obesity were not influential in affecting AscA levels in the infant. To date, the exact mechanism through which magnesium sulfate impacts on vitamin C levels remains unclear, with some suggesting that it enhances the expression of the sodium-dependent AscA transporter at the cellular level and increases cellular AscA uptake [43]. Our results found that preterm infant renal and hepatic maturational changes over time influenced AscA levels, suggesting that postnatal requirement of ascorbic acid may need to be augmented based on gestational maturity and/or renal and hepatic maturation.

We recognize that the subject sample size may limit the potential result, given possible sample bias and subsequently the external validity of the study, but to our knowledge, this is the largest sample collection and assessment of pAscA levels in a cohort of premature infants to date for assessing AscA changes over time, gestational maturity and during illness. We were also limited with the routine collection of cord-blood samples and day-one-of-life samples from all subjects enrolled in part due to the timing of and circumstances surrounding enrollment, despite efforts to keep consistency. Although our goal was to collect weekly samples until discharge, sample numbers per patient differed due to the expected difference in length of stay.

Ascorbic acid by nature is a very unstable molecule, with its degradation being accelerated in the presence of light, potentially leading to significantly lower measured levels than those truly representative of the actual state during growth and sickness. To reduce the amount of potential degradation and obtain samples most representative of the clinical scenario at time of collection, all samples were shielded from light using photoprotective sample-collection bags and stabilized in our lab for storage as quickly as possible. Additionally, laboratory standards were maintained to limit degradation. Current dietary vitamin recommendations are often overestimated and targeted to prevent certain pathologies no longer met in premature infants, as seen with ascorbic-acid deficiency and the prevention of scurvy, or in trying to mimic levels seen in utero. Our results suggest that the preterm infants rarely experienced deficiency levels that are currently in place. Instead, infants experienced commonly low “normal” levels, suggesting that current expected recommendations may need revision to higher dosing levels in the extremely preterm as well as potentially be increased during postnatal period in those infants most at risk for debilitating chronic lung disease. To more effectively and appropriately dose, supply and utilize AscA in premature infants, further studies are urgently needed to target better intake assessments and identify potential biomarkers of health to better understand appropriate dosing requirements of ascorbic acid during critical times of growth in regulating health and risk for inflammatory conditions in the vulnerable preterm infant.

## 5. Conclusions

Preterm infant pAscA levels vary based on maternal history of prenatal smoking, Mg SO4, infant birth gestational age and race. Lower pAscA levels during postnatal development are associated with risk of BPD and ROP in the infant ≤30 weeks. These results show prenatal and fetal developmental factors that influence preterm infants’ first pAscA levels with likely developmental-organ metabolism and body composition that may require nutritional ascorbic-acid-dosage changes during the stage for organ health development and risk pf disease in preterm infants.

## Figures and Tables

**Table 1:** Infant characteristics of study population (*n* = 63).

Infant Characteristics	
Gender, *n* (%)	
Female	31 (49.2)
Male	32 (50.8)
Race, *n* (%)	
Black	36 (57.1)
White	20 (31.8)
Hispanic	7 (11.1)
Gestational Age (weeks) ^a^	28.4 (3.1)
Birth Weight (g) ^a^	1092.4 (413.1)
Small for gestational age, *n* (%)	13 (20.6)

^a^ Continuous variable presented as mean (SD).

**Table 2:** Selected morbidities of study population (*n* = 63).

Infant Outcomes	
Length of Stay (days) ^a^	85.2 (50.4)
Bronchopulmonary Dysplasia, *n* (%)	
None	28 (44.4)
Mild	23 (36.5)
Moderate/Severe	12 (19.1)
Sepsis, *n* (%)	18 (28.6)
Any NEC, *n* (%)	9 (14)
Any IVH, *n* (%)	9 (14)
Any ROP, *n* (%)	36 (57)
Mortality, *n* (%)	6 (9)

^a^ Continuous variable presented as mean (SD). NEC = necrotizing enterocolitis, IVH = intraventricular hemorrhage, ROP = retinopathy of prematurity.

**Table 3:** Ascorbic-acid assessment of study population over time (*n* = 63).

Ascorbic-Acid Evaluations Mean (SD)	Week 1	>Week 1
Plasma AscA concentration (mg/dL) *	1.5 (0.72)	1.2 (0.8)
Day of life of plasma sample (days)	2 (1.8)	49 (35)
Urine AscA concentration (mg/dL)	17.8 (22)	23.8 (22)
Day of life of urine sample (days)	2 (2)	19 (24)
Creatinine at time of urine sample (mg/dL)	0.72 (0.15)	0.51 (0.25)

* Plasma ascorbic acid significantly decreased beyond the first week of life, regardless of corrected gestational age (1.5 mg/dL, SD (0.7) vs. 1.2 mg/dL, SD (0.8), *p* = 0.002).

**Table 4:** Early plasma ascorbic-acid levels and infant characteristics.

N = 55	Plasma Ascorbic-Acid Levels			
Characteristic	Difference	Std Error	95% Confidence Intervals	*p*-Value
Race				
Black vs. White	−0.44	0.20	(−0.90, 0.02)	0.0583
Black vs. Hispanic	0.25	0.26	(−0.35, 0.85)	0.3655
White vs. Hispanic	0.69	0.29	(0.02, 1.35)	0.0442
Gestational Age *	−0.16	0.06	(−0.30, −0.03)	0.0246

* Remained statistically significant after adjusting for race, SGA status and birthweight.

**Table 5:** Relationship between plasma ascorbic-acid levels during the first week of life and type of nutrition provided.

N = 53	Plasma Ascorbic-Acid Levels			
Characteristic	Difference	Std Error	95% Confidence Intervals	*p*-Value
Breastmilk Only				
No vs. Yes	0.49	0.36	(−0.36, 1.33)	0.2152
Formula Only				
No vs. Yes	0.57	0.71	(−1.12, 2.26)	0.4515
Breastmilk and Formula				
No vs. Yes	0.71	0.34	(−0.10, 1.52)	0.0774
TPN				
No vs. Yes	0.23	0.39	(−0.69, 1.16)	0.5682
Gestational Age *	−0.20	0.07	(−0.35, −0.04)	0.0185
Birthweight	0.001	0.00	(−0.00, 0.001)	0.1378
SGA Status				
No vs. Yes	0.31	0.38	(−0.59, 1.21)	0.4437

* Remained statistically significant after adjusting for type of nutrition provided, SGA status and birthweight.

**Table 6:** Patient characteristics ≤ 30 weeks (*n* = 47) vs. >30 weeks (*n* = 16).

Characteristics	≤30 Weeks	>30 Weeks
Gender, *n* (%)		
Female	21 (45)	10 (63)
Male	26(55)	6 (37)
Race, *n* (%)		
Black	32 (68)	4 (25)
White	10 (21)	10 (63)
Other	5 (11)	2 (12)
Gestational Age (weeks) ^a^	27.2 (2)	32 (1)
Birth Weight (g) ^a^	983 (302)	1543 (366)
SGA, *n* (%)	5 (11)	3 (18)
Length of Stay (days) ^a^	99 (48)	43 (30)
BPD Severity, *n* (%)		
None	14 (30)	14 (88)
Mild	16 (34)	2 (12)
Moderate/Severe	17 (36)	0
Sepsis, *n* (%)		
No	29 (62)	16 (100)
Yes	18 (38)	0
Plasma AscA concentration (mg/dL) ^a^	1.5 (0.74)	1.2 (0.7)
Day of life of sample (days) ^a^	6 (10)	13 (23)
Urine AscA concentration (mg/dL) ^a^	24 (22)	21 (26)
Day of life of sample (days) ^a^	20 (24)	14 (28)
Creatinine at time of urine sample (mg/dL) ^a^	0.56 (0.25)	0.65 (0.22)
AST (mg/dL) ^a^	59 (51)	50 (26)
ALT (mg/dL) ^a^	15 (38)	9 (9)
Direct Bilirubin (mg/dL) ^a^	0.6 (0.6)	1 (0.4)

^a^ Continuous variable presented as mean (SD).

**Table 7:** Plasma ascorbic-acid levels and risk for morbidities in ≤30 weeks premature infants.

N = 47	Mean pAscA (mg/dL)	95% Confidence Interval	*p*-Value
BPD Yes vs. No	1.3 ± SD vs. 2.0 ± SD	−0.36, 2.76	0.0003
ROP Yes vs. No	1.1 vs. 1.4	−0.43, 2.63	0.029
IVH Yes vs. No	1.0 vs. 1.2	−0.33, 1.72	0.23
Sepsis yes vs. No	1.2 vs. 1.2	−0.17, 2.57	0.7

BPD = bronchopulmonary dysplasia, ROP = retinopathy of prematurity, IVH = intraventricular hemorrhage, pAscA = plasma ascorbic acid.

## Data Availability

Not applicable.
